# Identification of Two Depolymerases From Phage IME205 and Their Antivirulent Functions on K47 Capsule of *Klebsiella pneumoniae*

**DOI:** 10.3389/fmicb.2020.00218

**Published:** 2020-02-14

**Authors:** Yannan Liu, Sharon Shui Yee Leung, Yong Huang, Yatao Guo, Ning Jiang, Puyuan Li, Jichao Chen, Rentao Wang, Changqing Bai, Zhiqiang Mi, Zhancheng Gao

**Affiliations:** ^1^Department of Respiratory and Critical Care Medicine, Peking University People’s Hospital, Beijing, China; ^2^School of Pharmacy, The Chinese University of Hong Kong, Shatin, Hong Kong; ^3^State Key Laboratory of Pathogen and Biosecurity, Beijing Institute of Microbiology and Epidemiology, Beijing, China; ^4^Department of Respiratory and Critical Care Medicine, The Fifth Medical Centre of Chinese PLA General Hospital, Beijing, China; ^5^Department of Respiratory and Critical Care Medicine, Aerospace Center Hospital, Beijing, China; ^6^Department of Respiratory and Critical Care Medicine, The First Medical Centre of Chinese PLA General Hospital, Beijing, China

**Keywords:** *Klebsiella pneumoniae*, carbapenem resistance, capsular type, capsule depolymerase, antivirulence

## Abstract

Carbapenem-resistant *Klebsiella pneumoniae* (CRKP) pose a significant threat to global public health. In present research, a total of 80 CRKP strains belonging to ST11 were collected with 70% (56 of 80 isolates) expressing a K47 capsular type. Thus, it is significant to prevent and control infections caused by these bacteria. Capsule depolymerases could degrade bacterial surface polysaccharides to reduce their virulence and expose bacteria to host immune attack. Previous studies have demonstrated the potential of phage-encoded depolymerases as antivirulent agents in treating CRKP infections *in vitro* and *in vivo*. Here, two capsule depolymerases (Dpo42 and Dpo43) derived from phage IME205 were expressed and characterized. Although both depolymerases act on strains with a capsular serotype K47, they are active against different subsets of strains, indicating subtle differences in capsule composition that exist within this serotype. The host range of phage IME205 matched to the sum of specificity range of Dpo42 and Dpo43. These two enzymes maintained stable activity in a relatively broad range of pH levels (pH 5.0–8.0 for Dpo42 and pH 4.0–8.0 for Dpo43) and temperatures (20–70°C). Besides, both Dpo42 and Dpo43 could make host bacteria fully susceptible to the killing effect of serum complement and display no hemolytic activity to erythrocytes. In summary, capsule depolymerases are promising antivirulent agents to combat CRKP infections.

## Introduction

*Klebsiella pneumoniae* is an opportunistic pathogen causing numerous hospital-acquired infections in immunocompromised individuals, including ventilator-associated pneumonia, catheter-related infection, urinary tract infection, soft tissue infection, surgical site infection and bacteremia (Paczosa and Mecsas, 2016). Moreover, this pathogen also causes community-acquired infections in young and healthy individuals, such as pyogenic liver abscesses (PLA), metastatic meningitis and endophthalmitis, presenting huge threats to global public health in the past few decades (Siu et al., 2012; Shon et al., 2013). With misuse and overuse of antibiotics, infections caused by multidrug-resistant (MDR) *K. pneumoniae* strains become an increasingly serious global problem. Among MDR isolates, carbapenem-resistant *K. pneumoniae* (CRKP) are of particular concern due to their extended antibiotic resistance phenotypes and ability to rapidly disseminate. Although the nosocomial infection and antimicrobial application have been effectively controlled, the proportions of CRKP isolates are still increasing (Grundmann et al., 2017; Heidary et al., 2018; Mao et al., 2019). Surveillance data in 2017 from China Antimicrobial Resistance Surveillance System (CARSS) indicated that about 20.9% and 24.0% of *K. pneumoniae* strains were resistant to imipenem and meropenem, respectively (Hu et al., 2018). As few available antibiotics can be used to treat CRKP infections, these strains are associated with high mortality. Thus, there is an urgent need to seek alternatives to conventional antibiotics against severe CRKP infections.

For most *K. pneumoniae* strains, capsule polysaccharides (CPS) are the major virulence factors that confer protective effects against phagocytosis and antimicrobials (Roach and Donovan, 2015). The glycan compositions of CPS which differ significantly between strains within the same bacterial species affect the bacterial virulence and allow the classification into distinct serotypes. Currently, a large number of capsular types (K-types) of *K. pneumoniae* have been described, including 77 types identified by serological reactivity tests and 5 new types (KN1–KN5) recognized by molecular genotyping (Pan et al., 2008, 2013, 2015, 2017; Hsu et al., 2013). Three new capsular types of CRKP isolates characterized by the primary structure of CPS were isolated from the outbreak or bloodstream infection (Kubler-Kielb et al., 2013; Bellich et al., 2019). Of these capsular types, K1 and K2 are associated with the emergence of hypervirulent *K. pneumoniae* (Follador et al., 2016). Pyogenic liver abscesses (PLA) caused by these bacteria have become a serious clinical challenge in many southeast Asian countries (Siu et al., 2012). K47 and K64 are the most prevalent types in MDR strains, particularly in CRKP infections in Asia (Pan et al., 2015; Gu et al., 2018; Huang et al., 2018; Liu et al., 2019c).

In recent years, capsule depolymerases were emerging as appealing antivirulence agents against infections caused by MDR bacteria. Unlike conventional antibiotics that kill bacterial cells or inhibit their growth, capsule depolymerases degrade bacterial surface polysaccharides to reduce their virulence and expose bacteria to host immune attack (Majkowska-Skrobek et al., 2016, 2018; Liu et al., 2019a, b; Oliveira et al., 2019). The potential of phage-encoded depolymerases of targeting various *K. pneumoniae* capsules have been demonstrated previously (Hsu et al., 2013; Lin et al., 2014; Pan et al., 2015, 2017, 2019; Majkowska-Skrobek et al., 2016, 2018; Hsieh et al., 2017; Cai et al., 2019; Wu et al., 2019). Recently, the depolymerase Dep42 encoded by phage SH-KP152226 could specifically degrade the K47 capsule of *K. pneumoniae* and was able to significantly inhibit biofilm formation or degrade formed biofilms (Wu et al., 2019). In the present study, we identified two depolymerases (Dpo42 and Dpo43) encoded by the same parent phage, IME205. The specificity ranges of these two enzymes were totally different, with the sum matching the host range of their parent phage. We suggest this was due to the subtle differences in capsule composition that exist within the K47 serotype. The enzymatic activities of these two proteins under different pH levels and temperatures were characterized. The serum sensitivity and hemolytic effect of them were also studied to confirm their antivirulence properties and safety for controlling severe CRKP infections. To the best of our knowledge, this is the first report of two depolymerases derived from a parent phage targeting different subsets of *K*. *pneumoniae* K47 capsules.

## Materials and Methods

### Strains Isolation and Antimicrobial Susceptibility Testing

A total of 80 clinical isolates of *K. pneumoniae* were collected from different inpatients between January 2018 and December 2018 at Aerospace Center Hospital, Beijing, China. The presumptive identification at the species level of *K. pneumoniae* strains was verified by *rpoB* gene sequencing (Drancourt et al., 2001; Rosenblueth et al., 2004; Saha et al., 2013). Of these strains, *K. pneumoniae* 94 and 256 showing hypermucoviscous phenotype were used to the characterization of Dpo42 and Dpo43. Antimicrobial susceptibility of these *K. pneumoniae* was determined using a Vitek 2.0 compact system (BioMerieux Clinical Diagnostics, Paris, France) to the following antimicrobial agents: ampicillin-sulbactam, piperacillin-tazobactam, cefazolin, cefuroxime, ceftriaxone, ceftazidime, cefepime, cefotetan, aztreonam, imipenem, meropenem, gentamicin, tobramycin, amikacin, trimethoprim-sulfamethoxazole, and ciprofloxacin. The minimum inhibitory concentrations (MICs) were determined by reference broth microdilution method and results were interpreted using the Clinical Laboratory Standard Institute breakpoints (Clinical and Laboratory Standards Institute [CLSI], 2018). *Pseudomonas aeruginosa* ATCC 27853 used as a quality control strain. In the present study, no human or animal subjects were involved, and therefore no ethics approval is required.

### Multilocus Sequence Typing (MLST) and Capsular Polysaccharide Serotyping

MLST of *K. pneumoniae* was performed according to Protocol 2 described on the MLST website^1^. The seven housekeeping genes (*gapA*, *infB*, *mdh*, *pgi*, *phoE*, *rpoB*, and *tonB*) of *K. pneumoniae* were amplified by PCR with primers in Protocol 2. Sequence types (STs) were determined by using the *K. pneumoniae* MLST database of Institut Pasteur^2^. To identify the capsular types of *K. pneumoniae* strains, the *wzi* allele was amplified, and the obtained sequences were aligned to the *wzi* sequences deposited in the database of Institut Pasteur (Brisse et al., 2013)^2^.

### Bacteriophage Isolation and Plaque Observation

The IME205 phage was isolated from a raw sewage sample collected at The Fifth Medical Centre of Chinese PLA General Hospital by using the hypermucoviscous *K. pneumoniae* 256 as host strain. Briefly, 4 mL of filtered sewage and 500 μL of bacteria culture in the exponential growth phase were added to 2 mL of 3 × Luria Bertani (LB) medium. After 4 h incubation at 37°C with shaking at 200 rpm, the culture was centrifuged and the supernatant was collected for double-layer agar plate assay to detect the presence of phage plaques (Kropinski et al., 2009).

### Phage DNA Extraction, Sequencing and Analysis

The phage nucleic acids were extracted using the High Pure Viral RNA Kit (Rocha Diagnostics, Mannheim, Germany) and sequenced using the Ion Personal Genome Machine System (Thermo Fisher Scientific, Waltham, MA, United States). The obtained reads were assembled into a complete genome sequence by the Newbler 2.9.1 software (Rocha Diagnostics, Bloomington, IN, United States). Open reading frames (ORFs) were annotated by using the Rapid Annotation in Subsystem Technology (RAST) web-server^3^, and the putative function of coding sequences (CDSs) was predicted by NCBI BLASTP.

### Expression, Purification, and Identification of Dpo42 and Dpo43

ORF42 (GenBank accession number ALT58497) and ORF43 (GenBank accession number ALT58498) were amplified by PCR with primers Dpo42p (5′-CAAATGGGTCGCGGATCCATGGACCAAGACATTAAAAC AG-3′ and 5′-GTGGTGGTGGTGCTCGAGTTACTGTTCGCC CCACTGCA-3′) and Dpo43p (5′-CAAATGGGTCGCGGATC ATGTTAAACAACCTGAACCAG-3′ and 5′-GTGGTGGTGG TGCTCGAGTTATGGACCAATAACCACACC-3′), respectively. The obtained PCR products were cloned into the pET28a vectors with restriction sites *Bam*HI and *Xho*I (New England Biolabs, Ipswich, MA, United States) using the *pEASY^®^* -Uni Seamless Cloning and Assembly Kit (TransGen Biotech, Beijing, China). The sequence of each insert in recombinant plasmids were checked by DNA sequencing before transformation into *E. coli* BL21(DE3). Cells carrying recombinant plasmids were cultured to the exponential phase and induced with 1 mM isopropyl β-D-1-thiogalactopyranoside (IPTG; Sigma-Aldrich, St. Louis, MO, United States) at 25°C overnight. After 5 min centrifugation at 10,000 rpm, the pellets were resuspended in lysis buffer (50 mM NaH_2_PO_4_, 300 mM NaCl, pH 8.0) and sonicated on ice (20 min with 3 s pulse and 4 s pause). Bacterial lysates were centrifuged and passed through a 0.45 μm filter. Then, the filtrates were loaded onto a gravity column with Ni-NTA resins (Sangon Biotech, Shanghai, China). The target protein was eluted with imidazole-containing buffer (50 mM NaH_2_PO_4_, 300 mM NaCl, 100 mM imidazole, pH 8.0) and dialyzed against a 1000-fold volume of lysis buffer over a 8–14 kDa molecular-mass-cutoff membrane (Viskase, Lombard, IL, United States). Protein molecular weight was estimated by 10% sodium dodecyl sulfate-polyacrylamide gel electrophoresis (SDS-PAGE), followed by Coomassie blue staining. The protein concentration was quantified with a fluorometer (Qubit 2.0, Thermo Fisher Scientific).

The Dpo42 or Dpo43 activity was qualitatively examined by the single-spot assay. Briefly, 500 μL of *K. pneumoniae* 94 or 256 culture in the exponential growth phase was mixed with 4.5 mL of molten soft nutrient agar to form a bacterial lawn on the plates. Then, 4 μL of diluted enzyme was dropped onto the plate and incubated overnight at 37°C. The enzymatic activity was monitored for the formation of halo zones.

### Specificity Range of Phage IME205, Dpo42, and Dpo43

The host range of phage IME205 was evaluated using the double-layer agar plate assay. Plates containing a mixture of IME205 and of each *Klebsiella* K47 strain were cultured overnight at 37°C. Phage-forming units (PFU) on bacterial lawns were counted. The experiment was independently conducted three times. Relative efficiency of plating (EOP) was calculated as the average PFU of the phage on the each tested bacteria divided by the average PFU on the host bacteria (*K. pneumoniae* 256) (Khan Mirzaei and Nilsson, 2015). The sensitivity of *Klebsiella* K47 strains to Dpo42 or Dpo43 (20 ng) was determined by the single-spot assay described above.

### Extraction of Bacterial Surface Polysaccharides

The extraction and purification of bacterial exopolysaccharides (EPS) containing both capsular polysaccharides and liposaccharides were performed via a modified hot water-phenol method as described previously (Hsieh et al., 2017). Briefly, 1 mL of *K. pneumoniae* 94 or 256 cultured overnight in LB with 0.25% glucose was centrifuged (10,000 rpm, 5 min) and resuspended in 200 μL of double distilled water (ddH_2_O). An equal volume of water-saturated phenol (pH 6.6; Thermo Fisher Scientific) was added to the bacterial suspension. The mixture was vortexed and incubated at 65°C for 20 min, then extracted with chloroform to remove bacterial debris. The obtained EPS was lyophilized and stored at −20°C.

### Quantification of Depolymerase Activity and Alcian Blue Staining

The enzymatic activity of Dpo42 or Dpo43 on bacterial surface polysaccharides was determined as described in Majkowska-Skrobek et al. (2016) with minor modifications. The EPS powder of *K. pneumoniae* 94 (or 256) was resuspended in ddH_2_O (2 mg/mL), and mixed with Dpo42 or Dpo43 (10 μg/mL) to a final reaction volume of 1.0 mL. EPS or enzyme alone served as positive or negative controls, respectively. After 1 h incubation at 37°C, cetylpyridinium chloride (CPC, Sigma-Aldrich) was added to the mixture (final concentration of 5 mg/mL) which was further incubated at room temperature (RT) for 5 min. Absorbance was measured at 600 nm using a Synergy HT Multi-Detection Microplate Reader (BioTek, VT, United States). The experiment was repeated at least three times.

The CPS was detected by Alcian blue staining as previously described (Pan et al., 2013; Hsieh et al., 2017). The treated samples were separated by a 10% SDS-PAGE. The gel was then washed for three different time duration (5, 10, and 15 min) with the fix/wash solution (25% ethanol, 10% acetic acid in water) and stained by 0.1% Alcian blue (Sigma-Aldrich) dissolved in the fix/wash solution for 15 min in the dark. CPS was visualized blue after the gel was destained overnight in the fix/wash solution.

### Influence of pH and Temperatures on the Depolymerase Activity

The EPS powder was dissolved in 100 mM citric acid-Na_2_HPO_4_ buffer (pH 3.0–8.0) or 100 mM Glycine-NaOH buffer (pH 9.0–10.0) to a final concentration of 2 mg/mL. The EPS solutions of *K. pneumoniae* 94 and 256 were mixed with Dpo42 and Dpo43 (10 μg/mL each) to a final reaction volume of 1.0 mL, respectively. After 1 h incubation at 37°C, the turbidity of residual EPS in various pH buffers was determined as described above. The effect of temperatures on the enzymatic activity was also determined. The EPS dissolved in citric acid-Na_2_HPO_4_ buffer (pH 5.0) was incubated with the depolymerase (10 μg/mL) at 20, 37, 50, 60, 70, 80, and 90°C for 1 h. EPS alone in citric acid-Na_2_HPO_4_ buffer (pH 5.0) at RT was served as the control. The enzymatic activity was determined as described above. The experiment was performed in triplicate.

### Serum Sensitivity

The serum sensitivity assay was conducted as described previously with slight modifications (Pan et al., 2015; Lin et al., 2017). Briefly, the human blood sample from healthy donor was centrifuged at 1,000 rpm for 10 min to obtain serum and erythrocytes. *K. pneumoniae* 94 and 256 were used to test the serum sensitivity with Dpo42 and Dpo43, respectively. Overnight bacteria cultures were centrifuged, resuspended in PBS to give a bacteria concentration of 10^9^ cfu/mL, and treated with the depolymerase at an enzyme concentration of 10 μg/mL. Then, the treated bacteria were immediately mixed with active or inactive serum (heated at 56°C for 30 min) at a volume ratio of 1:3 to a final reaction volume of 100 μL for 1 h incubation at 37°C. The untreated bacteria were incubated with enzyme or active serum as controls. The reaction mixtures were serially diluted and plated for bacterial counting. The experiment was independently performed three times.

### Hemolysis Assay

The hemolytic effect of depolymerases to erythrocytes was performed using previously described methods with minor modifications (Wang et al., 2015). The separated erythrocytes were washed and diluted to a concentration of 5% (v/v) with PBS. The erythrocytes were incubated with Dpo42 or Dpo43 (10 μg/mL) at 37°C for 1 h with gentle shaking at 60 rpm. The erythrocytes treated with PBS and 0.1% Triton X-100 were included as negative and positive control, respectively. After 10 min centrifugation at 1,000 rpm, 100 μL of supernatant was transferred to a 96-well microplate, and another 100 μL of PBS was added to the wells to a final volume of 200 μL. The amount of hemoglobin was measured at 540 nm. The experiment was conducted in triplicate.

### Statistical Analysis

All experimental data are presented as means ± standard deviation (SD), and the one-way analysis of variance (ANOVA) was used to compare multiple groups. The statistical analyses were conducted using Prism 7 (GraphPad Software, CA, United States), with *P* < 0.05 considered statistically significant.

## Results

### Capsular Types of *K. pneumoniae* and Plaque Characterization of IME205

**FIGURE 1:**
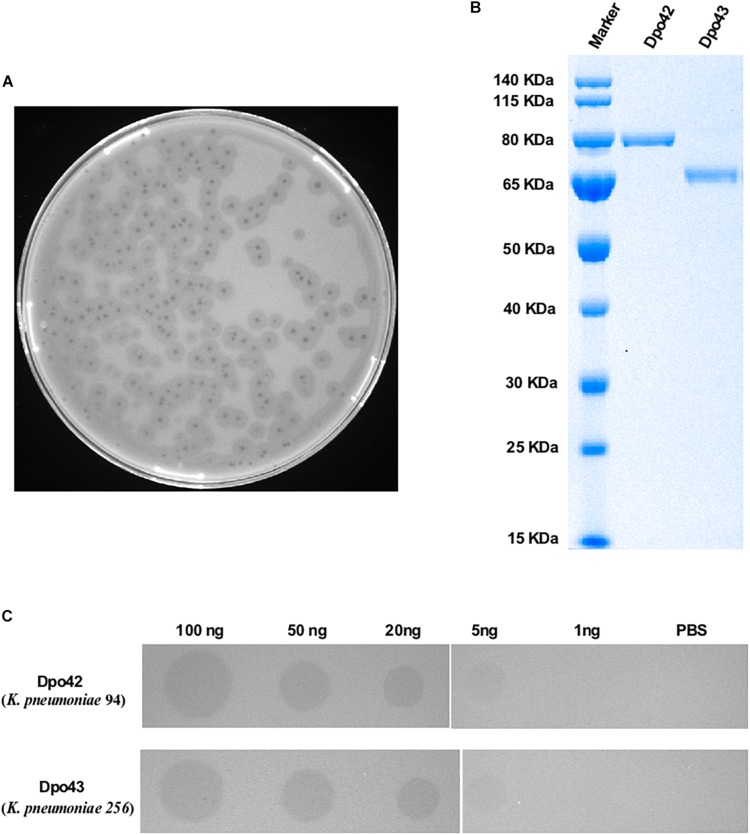
Plaque morphology of phage IME205 and characterization of depolymerases. **(A)** Phage IME205 produced clear plaques surrounded by translucent halos on the bacterial lawn of *K. pneumoniae* 256. **(B)** The molecular weight of purified Dpo42 and Dpo43 were determined by 10% SDS-PAGE with Coomassie blue staining. **(C)** The enzymatic activity was semi-quantified using serial dilutions of Dpo42 and Dpo43 on a lawn of *K. pneumoniae* 94 and 256, respectively. PBS served as negative controls.

During the study period, a total of 80 *K. pneumoniae* strains were identified to exhibit a carbapenem-resistant phenotype. The susceptibility rates of these bacteria toward different classes of antibiotics are given in Supplementary Table S1. These isolates were collected from sputum (66.25%, 53/80), urine (22.50%, 18/80), blood (6.25%, 5/80), wound secretion (2.50%, 2/80), anal swab (1.25%, 1/80) and central venous catheter (1.25%, 1/80). All strains belonged to ST11 and corresponded to four capsular types (Table 1), including K16 (1.25%, 1/80), K37 (2.50%, 2/80), K47 (70.00%, 56/80), and K64 (26.25%, 21/80). A lytic phage, IME205, was isolated using the *K. pneumoniae* 256 (K47) as the host bacterium. The phage formed clear plaques surrounded by translucent halos on the double-layer agar plate after overnight incubation at 37°C (Figure 1A). This observation suggested the phage IME205 produced depolymerases that could degrade bacterial surface polysaccharides.

**TABLE 1:** The capsular type of collected carbapenem-resistant *K. pneumoniae* belonging to ST11.

Serotype	Strain no.	Source	Percent (%)
K16	59	Urine	1.25(1/80)
K37	110	Sputum	2.50(2/80)
	180	Anal swab	
K47	04, 06, 07, 11, 12, 24, 26, 27, 28, 30, 43, 49, 72, 82, 84, 101, 104, 105, 108, 121, 157, 162, 168, 170, 174, 178, 189, 193, 196, 217, 220, 223, 228, 235, 248, 252	Sputum	70.00(56/80)
	60, 64, 76, 94, 114, 118, 126, 131, 146, 155, 186, 191, 231, 300	Urine	
	73	Wound secretion	
	134	Central venous catheter	
	194, 198, 254, 256	Blood	
K64	08, 13, 22, 44, 77, 83, 96, 103, 138, 159, 160, 188, 259, 262, 267, 303	Sputum	26.25(21/80)
	33, 42, 143	Urine	
	139	Wound secretion	
	268	Blood	

### ORF42 and ORF43 Had Depolymerase Activity

The obtained genome sequence of IME205 was deposited in GenBank under accession number KU183006. The complete genome of phage was 41,310 bp, with GC content of 52.2%. Online BLAST analysis showed that the complete genomic sequence of phage IME205 shares high similarity (90.67–98.39%) with that of 40 *Klebsiella* phages, all of which belong to *Podoviridae*. Whole-genomic comparison indicated that these phage genomes have similar structures, but the regions encoding phage tail fiber proteins are significantly different. The annotation results of RAST showed that the phage IME205 included 49 ORFs, and 30 of which were predicted to have specific functions by NCBI BLASTP (Supplementary Table S2). According to Latka et al. (2019), phage IME205 belongs to Group A of KP32viruses containing two receptor binding proteins (RBPs). A first gene encoding the phage tail fiber protein (ORF42) with a phage T7 tail fiber protein domain (residues 1–154) in the N-terminus (anchor-branch domain) and a pectate_lyase_3 protein with a predicted beta-helix structure (residues 300–502) in its central domain, and a second down-stream gene coding for a hypothetical protein (ORF43) with 83% identity to the second RBP of *Klebsiella* phage KP32 (GenBank accession number YP_003347556.1) are predicted to have polysaccharide depolymerase activities (Latka et al., 2019). The predicted length of ORF42 and OFR43 was 793 and 641 aa, respectively. The ORF42 and ORF43 were closest homologous to a tail fiber protein of *Klebsiella* phage IME304 (GenBank accession number QDB73379.1; identity = 99.50%, query cover = 100%) and a hypothetical protein of *Klebsiella* phage SH-KP152226 (GenBank accession number QDF14645.1; identity = 99.22%, query cover = 100%), respectively. Unfortunately, the capsular specificities of these two homologous proteins were not reported. To determine whether these CDSs had depolymerase activities, the PCR products of ORF42 and ORF43 were cloned into the pET28a vector, respectively. Then, proteins with a hexahistidine tag were expressed and purified. As depicted in Figure 1B, Dpo42 and Dpo43 migrated as single bands on 10% SDS-PAGE corresponding to their predicted molecular weight of 85 and 68 kDa, respectively. The concentration of purified Dpo42 and Dpo43 was determined to be 0.5 mg/mL and 0.4 mg/mL, respectively.

The depolymerase activity was determined using the modified single-spot assay, with enzyme dilutions ranging from 1 to 100 ng. As presented in Figure 1C, both Dpo42 and Dpo43 could form semi-clear halos on its host bacteria lawns. The size and transparency of semi-clear circles decreased with decreasing enzyme concentration, with the halo disappeared at a protein level of 1 ng.

### Specificity Range of Phage IME205, Dpo42, and Dpo43

The lytic spectrum of phage IME205 was determined among 56 strains of *Klebsiella* capsular type K47, including the host bacterium *K. pneumoniae* 256 (Table 2). Phage IME205 could produce plaques on all tested *K. pneumoniae* strains, with the EOP was greater than 0.1. Moreover, the sensitivity of these strains to Dpo42 or Dpo43 was also tested (Table 2). The single-spot assay showed a total of 16 *K. pneumoniae* strains (16/56) were sensitive to Dpo42, whereas the rest 40 strains (40/56) were sensitive to Dpo43.

**TABLE 2:** The specificity range of Dpo42, Dpo43 and their parent phage IME205.

Bacterial strains	IME205^a^	Dpo42 (ratio)^b^	Dpo43(ratio)^b^
04, 06, 07, 12, 24, 26, 30, 82, 84, 94, 121, 134, 146, 191, 196, 252	Y	Y (16/56)	N
11, 27, 28, 43, 49, 60, 64, 72, 73, 76, 101, 104, 105, 108, 114, 118, 126, 131, 155, 157, 162, 168, 170, 174, 178, 186, 189, 193, 194, 198, 217, 220, 223, 228, 231, 235, 248, 254, 256, 300	Y	N	Y (40/56)

*^*a*^The efficiency of plating (EOP) was greater than 0.1. ^*b*^The quantitation of Dpo42 or Dpo43 was 20 ng. Y, yes; N, no.*

### Dpo42 and Dpo43 Could Strip Bacterial Capsular Polysaccharides

**FIGURE 2:**
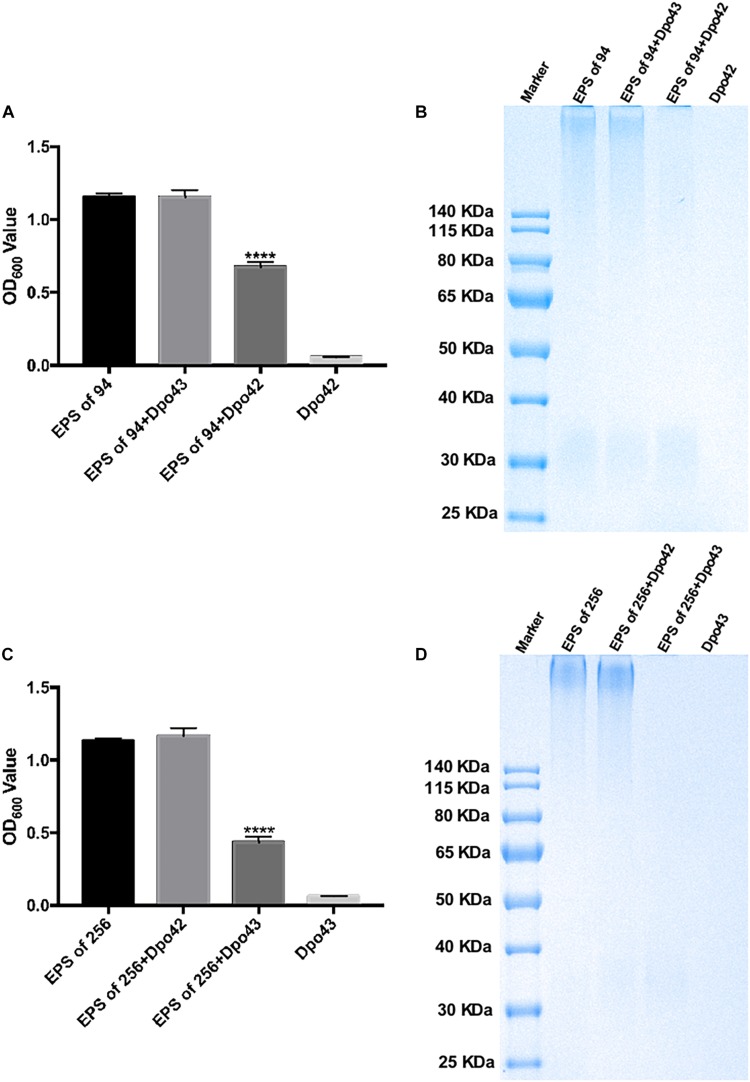
Capsule depolymerase activity of Dpo42 and Dpo43. The EPS extracted from *K. pneumoniae* 94 and 256 was treated with Dpo42 and Dpo43, respectively. EPS-alone and enzyme-alone served as positive and negative controls, respectively. **(A,C)** After 1 h incubation at 37°C, the CPC was added to the mixture for 5 min incubation at RT. The turbidity of residual EPS was measured at 600 nm. Data are presented as means ± SD (*n* = 6), and the statistical analysis was determined by one-way ANOVA (**** *P* < 0.0001). **(B,D)** The mixtures were separated by 10% SDS-PAGE, followed by Alcian blue staining.

The depolymerase activity was evaluated by monitoring the turbidity of the residual EPS. The EPS was extracted from *K. pneumoniae* 94 or 256, which belonged to different subsets of capsular type K47. As shown in Figures 2A,C, the OD_600_ values of EPS extracted from *K. pneumoniae* 94 were 1.156 ± 0.024, 1.154 ± 0.048, and 0.677 ± 0.033, after incubation with PBS, Dpo43 and Dpo42, respectively. For EPS extracted from *K. pneumoniae* 256, the OD_600_ values after treating with PBS, Dpo42 and Dpo43 were 1.133 ± 0.016, 1.167 ± 0.052, and 0.434 ± 0.038, respectively. The turbidity of enzyme alone was very low (0.058 ± 0.003 or 0.064 ± 0.002). This result revealed the turbidity of EPS treated with its specific enzyme was significantly decreased as compared with EPS alone or EPS incubated with its non-specific enzyme (*P* < 0.0001, one-way ANOVA). The capsule degrading properties of Dpo42 and Dpo43 were further verified by Alcian blue staining (Figures 2B,D). Results of the SDS-PAGE gel stained with Alcian blue showed that the CPS of *K. pneumoniae* 94 or 256 was apparently degraded by Dpo42 or Dpo43, respectively. The weight of EPS alone was almost the same as the EPS mixed with its non-specific enzyme, and the enzyme alone was not stained by Alcian blue.

### Activity of Dpo42 and Dpo43 Across a Range of pH Levels and Temperatures

**FIGURE 3:**
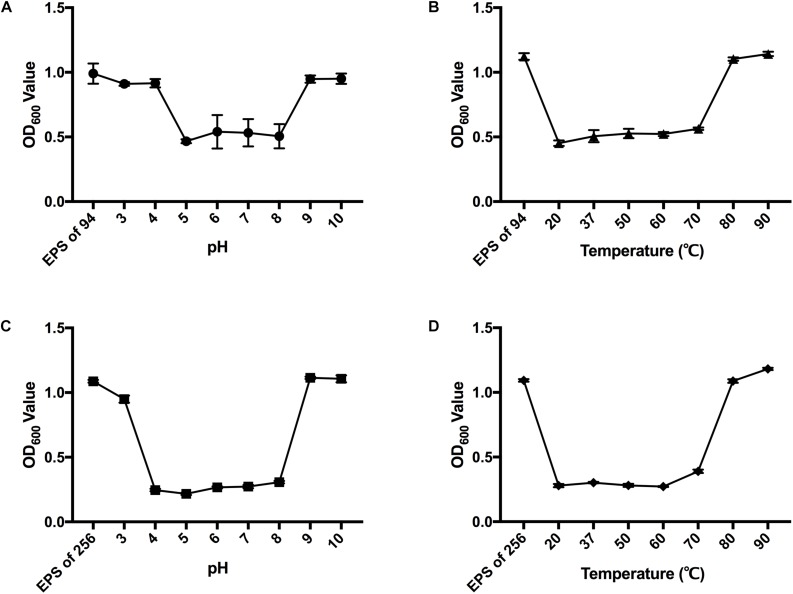
The effects of pH and temperature on the depolymerase activity. The EPS of *K. pneumoniae* 94 and 256 was mixed with Dpo42 **(A)** and Dpo43 **(C)**, respectively, with pH adjusted with 100 mM citric acid-Na_2_HPO_4_ buffer (pH 3.0–8.0) or 100 mM Glycine-NaOH buffer (pH 9.0–10.0) and incubated for 1 h at 37°C. The EPS of *K. pneumoniae* 94 and 256 was incubated with Dpo42 **(B)** and Dpo43 **(D)**, respectively, with pH adjusted to 5 and temperature varied between 20–90°C for 1 h. EPS-alone in citric acid-Na_2_HPO_4_ buffer (pH 5.0) at RT served as a control. The CPC was added to the mixture for 5 min incubation at RT. The turbidity of residual EPS was determined at 600 nm. Data are expressed as means ± SD (*n* = 3).

The enzymatic activity of Dpo42 or Dpo43 at pH 2 to 10 was determined (Figures 3A,C). The results showed that Dpo42 was active at pH 5.0 to 8.0, whereas the Dpo43 remained active at pH 4.0 to 8.0. The depolymerase activities of Dpo42 and Dpo43 were completely lost at pH outside their active ranges. The enzyme activities at temperatures ranging from 20 to 90°C in citric acid-Na_2_HPO_4_ buffer (pH 5.0) were determined (Figures 3B,D). Both Dpo42 and Dpo43 could maintain enzymatic activity at temperatures ranging from 20 to 70°C. The enzyme was completely inactive at 80°C.

### Depolymerase Sensitized Bacteria to Serum and Caused Non-hemolysis Reaction

**FIGURE 4:**
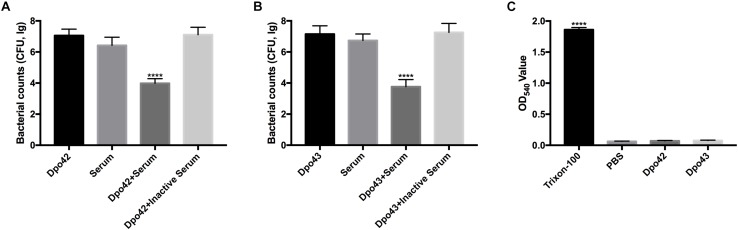
Serum killing and hemolysis assay. The overnight cultures of *K. pneumoniae* 94 and 256 were treated with Dpo42 **(A)** and Dpo43 **(B)**, respectively. The treated bacteria were immediately mixed with active or inactive serum (56°C, 30 min) at a volume ratio of 1:3, and the untreated bacteria were incubated with enzyme or active serum as a control. After 1 h incubation at 37°C, each mixture was serially diluted and plated for bacterial counting. **(C)** The erythrocytes were incubated with Dpo42 or Dpo43 at 37°C for 1 h with gentle shaking at 60 rpm, and the erythrocytes treated with PBS or 0.1% Triton X-100 as a control. The hemoglobin in supernatant was quantified at 540 nm. The values are indicated as means ± SD (*n* = 6), and the one-way ANOVA was conducted to compare multiple groups (**** *P* < 0.0001).

To confirm Dpo42 or Dpo43 could sensitize *K. pneumoniae* to serum, the viable counts of bacteria in various treatment conditions were determined. The *K. pneumoniae* 94 and 256 were sensitize to Dpo42 and Dpo43, respectively. Figures 4A,B showed that the number of bacteria treated with its specific enzyme followed with serum was effectively decreased (≥3 log reduction) as compared with other groups (*P* < 0.0001, one-way ANOVA). While the serum alone slightly reduced the bacterial counts, the enzyme alone and the enzyme mixed with inactive serum did not affect the bacterial survival. Thus, both Dpo42 and Dpo43 could make bacteria more susceptible to the serum complement-mediated killing. To assess the safety of applying Dpo42 and Dpo43 as antimicrobial agents, the hemolysis of enzyme toward erythrocytes was assessed *in vitro* (Figure 4C). The Dpo42 and Dpo43 displayed no hemolytic activity to erythrocytes.

## Discussion

In 2013, the US Centers for Diseases Control and Prevention (CDC) reported the emergence of carbapenem-resistant Enterobacteriaceae infections poses an urgent threat to public health. Among the carbapenem-resistant Enterobacteriaceae pathogens, the CRKP isolates accounted for 70–90% of clinical infections in China (Zhang et al., 2017). Furthermore, most clinical CRKP strains belonged to clonal group 258, with ST11 being the most prevalent clone in Asia and accounting for more than 60% of CRKP in China (Chen et al., 2014; Zhang et al., 2017). In 2018, five patients died due to severe lung infection, multi-organ failure, or septic shock caused by carbapenem-resistant hypervirulent *K. pneumoniae* strains belonging to ST11 and serotype K47 in the Second Affiliated Hospital of Zhejiang University (Hangzhou, China) (Gu et al., 2018). Besides, an extensively drug-resistant (XDR) and carbapenemase-producing hypervirulent strain belonging to ST11 and capsular type K47 was isolated from a 83-year-old female patient, who died from intra-abdominal abscess in Taiwan (Huang et al., 2018). Therefore, it is significant to effectively diagnose and treat CRKP infections, especially those belonging to ST11 and capsular type K47.

Here, we collected a total of 80 CRKP isolates belonging to ST11 from different inpatients and identified their capsular types (Table 1). Up to 96.25% of *K. pneumoniae* strains belonged to capsular type K47 (70.00%) and K64 (26.25%), in accordance with other findings (Pan et al., 2015; Gu et al., 2018; Huang et al., 2018; Liu et al., 2019c). Recently, phage-encoded depolymerases have shown great potential as antivirulent agents in treating CRKP infections *in vitro* and *in vivo* studies, including enzymes which could degrade *K. pneumoniae* K47 or K64 capsules (Pan et al., 2015, 2017; Wu et al., 2019). These proteins showed no side effects on normal microbiota and low incidence of resistance to target bacteria (Liu et al., 2019a, b). In the present study, we aimed to identify two depolymerases (Dpo42 and Dpo43) encoded by a parent phage that are active against *K*. *pneumoniae* capsular type K47 due to the high prevalence rate in the Asia region.

We first isolated a lytic phage IME205 that can infect all the collected *K. pneumoniae* strains of capsular type K47 (a total of 56 strains). Through analyzing the phage genome, we found that the phage tail fiber protein (ORF42) had a phage T7 tail fiber protein domain (residues 1–154) in the N-terminus and a pectate_lyase_3 protein with a predicted beta-helix structure (residues 300–502) in its central domain, which was previously shown to be responsible for decapsulation of bacteria (Pires et al., 2016). The single-spot assay confirmed that the recombinant enzyme (Dpo42) had depolymerase activity to a portion of the collected K47 *Klebsiella* strains (16/56), with the rest (40/56) being non-specific to this protein (Table 2). As the phage IME205 could also form plaques with translucent halos on these non-specific bacteria, we speculated this phage might encode another protein with depolymerase activity. Earlier studies indicated that the up-stream or down-stream genes of tail fiber protein could function as polysaccharide depolymerases (Hsieh et al., 2017; Majkowska-Skrobek et al., 2018). Besides, the down-stream gene of ORF42 was 83% identical to the second RBP of *Klebsiella* phage KP32 (GenBank accession number YP_003347556.1) (Latka et al., 2019). Therefore, we cloned and expressed the sequence of ORF43 and found this protein (Dpo43) was active against the rest K47 *K. pneumoniae* strains which were non-specific to Dpo42. This result was in agreement with previous reports that the lytic spectrum of a phage often matched with the sum of specificity range of individual depolymerases (Hsieh et al., 2017; Pan et al., 2017; Majkowska-Skrobek et al., 2018).

It is worth noting that this is the first report showing two depolymerases target different range of *K. pneumoniae* strains of capsular type K47. In this research, the *wzi* sequence of each *K. pneumoniae* K47 type was 100% identical to the reference *wzi* sequence deposited in the database of Institute Pasteur. However, according to Brisse et al. (2013), the K type cannot always be deduced based on *wzi* given sequence identities of some strains with distinct K types. It is because the K-type reference strains are old and poorly represent the current population of *K. pneumoniae* clinical isolates. Therefore, the *wzc* sequences were also used to identify the capsular types of these strains (Pan et al., 2013). The results indicated that these 56 *K. pneumoniae* strains had the same sequences in the variable regions of *wzc* sequences, which were closest homologous to *K. pneumoniae* K47 type. The reason of these two enzymes specified to different subsets of *K. pneumoniae* K47 type might be attributed to the subtle difference in sugar compositions of the bacterial capsules, though these test *K. pneumoniae* strains had the same capsular genotype. In other words, the genotype analysis might miss subtle changes in sugar compositions of the bacterial surface (Pan et al., 2019). Both *wzi* and *wzc* sequences of *K*. *pneumoniae* K47 capsules were provided in Supplementary Data Sheets S1, S2.

The activity of the two proteins was stable under moderate acidic or alkaline conditions (pH 5.0–8.0 for Dpo42 and pH 4.0–8.0 for Dpo43), similar to other depolymerases encoded by *Klebsiella* KP36 Phage (Majkowska-Skrobek et al., 2016). On the contrary, the activity of enzyme derived from *Klebsiella* phage KP32 or targeted to *K. pneumoniae* B5055 found to be stable under slightly alkaline conditions from pH 7.0–9.0 or 6.0–10.0, respectively (Kassa and Chhibber, 2012; Majkowska-Skrobek et al., 2018). Besides, both Dpo42 and Dpo43 could maintain enzymatic activity at temperatures ranging from 20 to 70°C (Figures 3B,D), in line with most *Klebsiella* depolymerases (Kassa and Chhibber, 2012; Majkowska-Skrobek et al., 2016, 2018). Therefore, both Dpo42 and Dpo43 were stable under the physiological conditions to exert its antivirulent capability during the therapy.

Depolymerases could strip bacterial capsules and thus sensitize bacteria to serum killing (Lin et al., 2014; Pan et al., 2015). In the serum killing assay, bacteria grown overnight were treated with enzyme and serum to mimic treatment of severe infections *in vivo*. After 1 h incubation at 37°C, the viable counts of bacteria treated with enzyme and serum mixture was effectively decreased (≥3 log reduction) as shown in Figures 4A,B. On the other hand, the enzyme mixed with inactive serum scarcely affected the bacterial survival. This result suggested that the serum complement played an important role in serum killing assays, consistent with other findings (Lin et al., 2017; Majkowska-Skrobek et al., 2018). It was noted that the enzyme mixed with serum could not completely eradicate all bacteria. Similar observation was noted in our previous study (Liu et al., 2019b). We found that Dpo48 depolymerase could form translucent halos on the lawn of non-treated bacteria but not on the lawn of enzyme-treated bacteria, suggesting that the enzyme should have removed the capsules of the bacteria. We also tested the effect of increasing the ratio of serum volume on the killing efficiency of enzyme-pretreated bacteria and no significant enhancement was noted. Therefore, we speculated the incomplete bacteria eradication might attributed to the presence of a subpopulation of bacteria which were susceptible to the depolymerases but resist serum complement-mediated killing. To develop the enzyme as therapeutic agents, it is important to ensure their safety. Both Dpo42 and Dpo43 displayed no hemolytic activity to erythrocytes. Thus, these two proteins could consider to be further studied as therapeutic agents *in vivo*.

## Conclusion

Depolymerases Dpo42 and Dpo43 derived from phage IME205 could degrade *K. pneumoniae* capsules of K47. Their specificity ranges were completely different, but their sum match with the host range of their parental phage. Both enzymes could maintain their depolymerase activities over a broad range of pH levels and temperatures, allowing them to be applied in physiological conditions. They could effectively sensitize bacteria to serum complement-mediated killing and exhibited no hemolytic activity to erythrocytes. These promising results warrant further studies to develop these two depolymerases as an alternative treatment for prevention and control of severe infections caused by CRKP.

## Data Availability Statement

The datasets generated for this study can be found in the GenBank under accession number KU183006.

## Author Contributions

YL conceived the project, performed the biological experiments, analyzed the data, and wrote the manuscript. SL helped revise and edit manuscript. YH assembled the reads into a complete genome sequence. YG, NJ, and JC collected clinical isolates. PL and RW helped prepare some figures and tables. CB, ZM, and ZG helped conceive the project and contributed reagents or materials. All authors reviewed the manuscript.

## Conflict of Interest

The authors declare that the research was conducted in the absence of any commercial or financial relationships that could be construed as a potential conflict of interest.

## Footnotes

1 http://bigsdb.pasteur.fr/klebsiella/primers_used.html

2 http://bigsdb.pasteur.fr/klebsiella/klebsiella.html

3 http://rast.nmpdr.org/

## Supplementary Material

TABLE S1 Susceptibility rates toward different classes of antibiotics for *K. pneumoniae* strains belonging to ST11.

TABLE S2 The list of ORFs in the genome of phage IME205 and their putative functions.

DATA SHEET S1 The *wzi* sequences of *K. pneumoniae* K47 capsules.

DATA SHEET S2 The *wzc* sequences of *K. pneumoniae* K47 capsules.
