# Supplementary material for: Identification of Two Depolymerases From Phage IME205 and Their Antivirulent Functions on K47 Capsule of Klebsiella pneumoniae
Source: Front Microbiol. 2020 Feb 14;11:218. doi: 10.3389/fmicb.2020.00218 (PMC7034173; doi:10.3389/fmicb.2020.00218)
Supplement: TABLE S1 — Susceptibility rates toward different classes of antibiotics for K. pneumoniae strains belonging to ST11. [file Table_1.DOCX]

**Supplementary Table 1. Susceptibility rates toward different classes of antibiotics for *K. pneumoniae* strains belonging to ST11**

| **Antimicrobial category** | **Antimicrobial agent** | **Susceptible (%)** | **Intermediate (%)** | **Resistant (%)** |
| --- | --- | --- | --- | --- |
| Penicillins + *β*-lactamase inhibitors | Ampicillin-sulbactam | 0 | 0 | 100 |
| Antipseudomonal penicillins +*β*-lactamase inhibitors | Piperacillin-tazobactam | 0 | 1.25 (1/80) | 98.75 (79/80) |
| Non-extended spectrum cephalosporins | Cefazolin | 0 | 0 | 100 |
|  | Cefuroxime | 0 | 0 | 100 |
| Extended-spectrum cephalosporins | Ceftriaxone | 0 | 0 | 100 |
|  | Ceftazidime | 0 | 1.25 (1/80) | 98.75 (79/80) |
|  | Cefepime | 2.50 (2/80) | 0 | 97.50 (78/80) |
| Cephamycins | Cefotetan | 3.75 (3/80) | 2.50 (2/80) | 93.75 (75/80) |
| Monobactams | Aztreonam | 0 | 0 | 100 |
| Carbapenems | Imipenem | 0 | 0 | 100 |
|  | Meropenem | 0 | 1.25 (1/80) | 98.75 (79/80) |
| Aminoglycosides | Gentamicin | 33.75 (27/80) | 1.25 (1/80) | 65.00 (52/80) |
|  | Tobramycin | 38.75 (31/80) | 6.25 (5/80) | 55.00 (44/80) |
|  | Amikacin | 62.50 (50/80) | 2.50 (2/80) | 35.00 (28/80) |
| Folate pathway inhibitors | Trimethoprim-sulphamethoxazole | 53.75 (43/80) | 0 | 46.25 (37/80) |
| Fluoroquinolones | Ciprofloxacin | 0 | 0 | 100 |
