# Supplementary material for: Identification of Two Depolymerases From Phage IME205 and Their Antivirulent Functions on K47 Capsule of Klebsiella pneumoniae
Source: Front Microbiol. 2020 Feb 14;11:218. doi: 10.3389/fmicb.2020.00218 (PMC7034173; doi:10.3389/fmicb.2020.00218)
Supplement: TABLE S2 — The list of ORFs in the genome of phage IME205 and their putative functions. [file Table_2.DOCX]

**Supplementary Table 2. The list of ORFs in the genome of phage IME205 and their putative functions.**

| **ORF** | **Location (nt)^a^** | **Product**  **Length (aa)^b^** | **Homologue** | **Accession number** | **Sequence**  **query cover (%)^c^** | **Sequence**  **identity (%)^c^** |
| --- | --- | --- | --- | --- | --- | --- |
| 1 | 899..1237 | 112 | protein kinase of Klebsiella phage kpssk3 | ALT58456 | 95 | 92.52 |
| 2 | 1230..1376 | 48 | hypothetical protein of Klebsiella phage K5-2 | ALT58457 | 100 | 75 |
| 3 | 1354..1551 | 65 | hypothetical protein of Klebsiella virus KP32 | ALT58458 | 100 | 96.92 |
| 4 | 1535..1705 | 56 | hypothetical protein of Klebsiella Henu1 | ALT58459 | 100 | 83.93 |
| 5 | 1733..2746 | 337 | protein kinase of Klebsiella phage kpssk3 | ALT58460 | 100 | 90.09 |
| 6 | 2816..5536 | 906 | DNA-directed RNA polymerase of Escherichia phage K30 | ALT58461 | 100 | 99.12 |
| 7 | 5635..5811 | 58 | hypothetical protein of Klebsiella phage K5 | ALT58462 | 100 | 100 |
| 8 | 5815..6081 | 88 | hypothetical protein of Escherichia phage K30 | ALT58463 | 100 | 96.59 |
| 9 | 6162..6590 | 142 | hypothetical protein of Klebsiella phage vB_KpnP_BIS33 | ALT58464 | 100 | 97.18 |
| 10 | 6587..7645 | 352 | DNA ligase of Klebsiella phage K5-2 | ALT58465 | 100 | 92.05 |
| 11 | 7763..8026 | 87 | hypothetical protein of Enterobacter phage phiEap-1 | ALT58466 | 100 | 90.80 |
| 12 | 8019..8405 | 128 | hypothetical protein of Klebsiella phage SH-Kp 152410 | ALT58467 | 99 | 96.85 |
| 13 | 8395..8520 | 41 | hypothetical protein of Klebsiella phage K11 | ALT58468 | 100 | 95.12 |
| 14 | 8486..8635 | 49 | host RNA polymerase inhibitor of Klebsiella virus KP32 | ALT58469 | 100 | 100 |
| 15 | 8695..9390 | 231 | single-stranded DNA-binding protein of Escherichia phage K30 | ALT58470 | 100 | 99.57 |
| 16 | 9390..9839 | 149 | endonuclease I of Klebsiella phage KP32 | ALT58471 | 100 | 100 |
| 17 | 9842..10297 | 151 | N-acetylmuramoyl-L-alanine amidase of Klebsiella phage KN3-1 | ALT58472 | 100 | 99.34 |
| 18 | 10774..11088 | 104 | homing endonuclease of Klebsiella virus KP32 | ALT58473 | 100 | 96.15 |
| 19 | 11306..12832 | 508 | primase/helicase protein of Klebsiella phage vB_KpnP_IME321 | ALT58474 | 100 | 99.61 |
| 20 | 12898..13107 | 69 | hypothetical protein of Enterobacter phage phiEap-1 | ALT58475 | 100 | 60.29 |
| 21 | 13107..13421 | 104 | hypothetical protein of Klebsiella virus KP32 | ALT58476 | 100 | 91.35 |
| 22 | 13491..13922 | 143 | HNH endonuclease of Enterobacteria phage BA14 | ALT58477 | 100 | 67.83 |
| 23 | 13916..16042 | 708 | DNA polymerase of Klebsiella phage 2044-307w | ALT58478 | 100 | 97.88 |
| 24 | 16061..16348 | 95 | HNS binding protein of Klebsiella phage vB_Kp1 | ALT58479 | 100 | 100 |
| 25 | 16345..16554 | 69 | hypothetical protein of Klebsiella phage K5 | ALT58480 | 100 | 98.55 |
| 26 | 16563..16727 | 54 | hypothetical protein of Klebsiella phage vB_KpnP_BIS33 | ALT58481 | 100 | 100 |
| 27 | 16720..17625 | 301 | exonuclease of Klebsiella phage vB_Kp1 | ALT58482 | 100 | 97.67 |
| 28 | 17803..18048 | 81 | hypothetical protein of Klebsiella phage K11 | ALT58483 | 100 | 100 |
| 29 | 18051..18272 | 73 | hypothetical protein of Klebsiella phage K11 | ALT58484 | 100 | 97.26 |
| 30 | 18274..18534 | 86 | tail assembly protein of Klebsiella phage K11 | ALT58485 | 100 | 100 |
| 31 | 18546..20153 | 535 | head-to-tail joining protein of Klebsiella phage K11 | ALT58486 | 100 | 99.25 |
| 32 | 20257..21213 | 318 | capsid and scaffold protein of Klebsiella phage K5-4 | ALT58487 | 100 | 96.86 |
| 33 | 21447..22478 | 343 | major capsid protein of Klebsiella phage Henu1 | ALT58488 | 100 | 99.42 |
| 34 | 22535..22756 | 73 | hypothetical protein of Klebsiella phage vB_KpnP_KpV289 | ALT58489 | 100 | 97.26 |
| 35 | 22823..23401 | 192 | tail tubular protein A of Klebsiella virus KP32 | ALT58490 | 100 | 99.48 |
| 36 | 23424..25799 | 791 | tail tubular protein B of Klebsiella virus KP32 | ALT58491 | 100 | 98.48 |
| 37 | 25872..26282 | 136 | internal virion protein A of Escherichia phage K30 | ALT58492 | 100 | 100 |
| 38 | 26355..26660 | 101 | endonuclease VII of Klebsiella phage kpssk3 | ALT58493 | 100 | 92.08 |
| 39 | 26660..27250 | 196 | internal virion protein B of Klebsiella phage SH-Kp 152410 | ALT58494 | 100 | 97.45 |
| 40 | 27250..29505 | 751 | internal virion protein C of Escherichia phage K30 | ALT58495 | 100 | 98.93 |
| 41 | 29522..33487 | 1321 | internal virion protein D of Klebsiella phage vB_Kp1 | ALT58496 | 100 | 99.02 |
| 42 | 33550..35931 | 793 | tail fiber protein of Enterobacter phage phiEap-1 | ALT58497 | 99 | 41.18 |
| 43 | 35950..37875 | 641 | hypothetical protein of Escherichia coil | ALT58498 | 99 | 45.75 |
| 44 | 37885..38088 | 67 | holin of Klebsiella phage K11 | ALT58499 | 100 | 100 |
| 45 | 38092..38346 | 84 | DNA packaging protein A of Klebsiella phage vB_KpnP_PRA33 | ALT58500 | 100 | 98.81 |
| 46 | 38406..38522 | 38 | hypothetical protein of Klebsiella phage vB_KpnP_PRA33 | ALT58501 | 100 | 89.47 |
| 47 | 38532..38978 | 148 | endopeptidase of Klebsiella phage K5 | ALT58502 | 100 | 93.92 |
| 48 | 38975..40732 | 585 | DNA packaging protein B of Escherichia phage K30 | ALT58503 | 100 | 99.49 |
| 49 | 40977..41126 | 49 | hypothetical protein of Klebsiella phage vB_Kp1 | ALT58504 | 100 | 97.96 |

^a^ nt, nucleotide; ^b^ aa, amino acid; ^c^ determined by BLAST-P.
